# Suppression of angiotensin II-induced pathological changes in heart and kidney by the caveolin-1 scaffolding domain peptide

**DOI:** 10.1371/journal.pone.0207844

**Published:** 2018-12-21

**Authors:** Panneerselvam Chinnakkannu, Charles Reese, John Antony Gaspar, Saraswathi Panneerselvam, Dorea Pleasant-Jenkins, Rupak Mukherjee, Catalin Baicu, Elena Tourkina, Stanley Hoffman, Dhandapani Kuppuswamy

**Affiliations:** 1 Division of Cardiology, Department of Medicine, Gazes Cardiac Research Institute, Medical University of South Carolina, Charleston, South Carolina, United States of America; 2 Division of Rheumatology, Department of Medicine, Medical University of South Carolina, Charleston, South Carolina, United States of America; 3 Research Centre, Ananda College, Devakottai, Tamil Nadu, India; 4 Department of Regenerative Medicine and Cell Biology, Medical University of South Carolina, Charleston, South Carolina, United States of America; 5 Division of Cardiothoracic Surgery, Department of Surgery, Medical University of South Carolina, Charleston, South Carolina, United States of America; Max Delbruck Centrum fur Molekulare Medizin Berlin Buch, GERMANY

## Abstract

Dysregulation of the renin-angiotensin system leads to systemic hypertension and maladaptive fibrosis in various organs. We showed recently that myocardial fibrosis and the loss of cardiac function in mice with transverse aortic constriction (TAC) could be averted by treatment with the caveolin-1 scaffolding domain (CSD) peptide. Here, we used angiotensin II (AngII) infusion (2.1 mg/kg/day for 2 wk) in mice as a second model to confirm and extend our observations on the beneficial effects of CSD on heart and kidney disease. AngII caused cardiac hypertrophy (increased heart weight to body weight ratio (HW/BW) and cardiomyocyte cross-sectional area); fibrosis in heart and kidney (increased levels of collagen I and heat shock protein-47 (HSP47)); and vascular leakage (increased levels of IgG in heart and kidney). Echocardiograms of AngII-infused mice showed increased left ventricular posterior wall thickness (pWTh) and isovolumic relaxation time (IVRT), and decreased ejection fraction (EF), stroke volume (SV), and cardiac output (CO). CSD treatment (i.p. injections, 50 μg/mouse/day) of AngII-infused mice significantly suppressed all of these pathological changes in fibrosis, hypertrophy, vascular leakage, and ventricular function. AngII infusion increased β1 and β3 integrin levels and activated Pyk2 in both heart and kidney. These changes were also suppressed by CSD. Finally, bone marrow cell (BMC) isolated from AngII-infused mice showed hyper-migration toward SDF1. When AngII-infused mice were treated with CSD, BMC migration was reduced to the basal level observed in cells from control mice. Importantly, CSD did not affect the AngII-induced increase in blood pressure (BP), indicating that the beneficial effects of CSD were not mediated via normalization of BP. These results strongly indicate that CSD suppresses AngII-induced pathological changes in mice, suggesting that CSD can be developed as a treatment for patients with hypertension and pressure overload-induced heart failure.

## Introduction

Metabolic syndromes such as hypertension, obesity, and/or diabetes mellitus often cause organ fibrosis, primarily affecting the heart and kidney and contribute to the development of congestive heart failure (CHF). Elevated angiotensin-II (AngII) causes hypertension that results in endothelial dysfunction resulting in impaired vasodilation, vascular inflammation due to leucocyte-endothelial cell interaction, and increased vascular permeability [1]. The activation of angiotensin receptor 1 (AT1) by AngII also mediates the development of cardiac and renal fibrosis [2–4] by stimulating synthesis of proinflammatory cytokines, chemokines, adhesion molecules, and growth factors that in turn activate their cognate receptors to promote the proliferation and differentiation of fibroblasts into myofibroblasts that express extracellular matrix (ECM) proteins at high levels [5]. These processes involve AngII upregulation of TGFβ expression in both cardiomyocytes and cardiac fibroblasts and the resulting contributions of AngII and TGFβ signaling to fibrosis and cardiac hypertrophy as part of pathological ventricular remodeling [4].

Myocardial fibrosis is a major pathological feature associated with ventricular remodeling in patients with congestive heart failure (CHF) [6–8]. It impairs cardiac compliance by increasing ventricular wall stiffness and reducing oxygen diffusion and electrical coupling leading to compromised diastolic and systolic functions. Unlike reparative fibrosis, which is essential for myocardial infarct healing subsequent to cardiomyocyte loss; interstitial fibrosis, as occurs due to increased levels of AngII, proceeds mostly as a maladaptive response and compromises ventricular performance. Recent studies point to cardiac fibroblasts as the major cell type that contributes to myocardial fibrosis and the associated maladaptive remodeling of chronic PO myocardium [6, 9, 10]. Although fibroblasts are considered to be the primary cell type responsible for collagen turnover [10, 11], the origin of these cells during organ fibrosis remains controversial [3, 12]. Several studies show that fibrosis results from the activation of resident fibroblasts [13, 14] by cytokines produced by inflammatory cells, particularly macrophages recruited into the injured tissue [15]. However, cells of intermediate phenotypes between monocytes and fibroblasts (i.e., referred to as fibrocytes) [16–19] are present at high levels in hypertrophic cardiomyopathy [5, 20–26], suggesting that the hematopoietic lineage contributes to fibroblasts present in fibrotic disease. Furthermore, other independent studies show proliferating fibroblast-like cells near blood vessels, suggesting the possible role of endothelial cells and pericytes as precursors of myofibroblasts [27, 28].

Increased levels of AngII are also known to affect renal perfusion, cause kidney fibrosis, and decrease glomerular function [2, 29]. Decreased renal perfusion releases renin in the kidney that cleaves angiotensinogen to angiotensin I. Angiotensin-converting enzyme (ACE) converts angiotensin I to AngII causing a further increase in AngII levels [30]. Therefore, AngII is an important mediator in the progression of both renal and myocardial fibrosis, and ACE inhibitors have been shown to have beneficial effects in patients with heart, kidney, and other organ fibrosis [2, 31, 32].

Studies involving fibrosis in other organs have demonstrated that a deficiency of the caveolae structural protein caveolin-1 in monocytes and fibroblasts contributes to organ fibrosis [33–38] and that administration of the caveolin-1 scaffolding domain peptide (CSD, a 20-amino acid segment of caveolin-1 that enters cells and acts as a functional surrogate) can block the development of organ fibrosis [35–37, 39–43].

In earlier studies, we used a transverse aortic constriction (TAC) mouse model to demonstrate that the activation of β3 integrin and nonreceptor tyrosine kinases (NTKs such as c-Src and Pyk2) play critical roles in pressure overload-induced myocardial fibrosis [44, 45]. Furthermore, TAC-induced β3 integrin/NTKs signaling, myocardial fibrosis, and compromised ventricular function were substantially reduced when mice were treated with CSD [43]. To explore CSD’s effect in other models of organ fibrosis, we used an AngII infusion mouse model to study the antifibrotic effect of CSD in both the heart and kidney. Our present work shows that AngII infusion for 2 wk results in the development of heart and kidney fibrosis with increased levels of collagen I and HSP47. AngII-induced effects were substantially reduced in mice treated with CSD. Further, AngII infusion caused cardiac hypertrophy with increased LV mass and pWTh with compromised ventricular function, vascular leakage resulting in the release of IgG into heart and kidney, and hyper-migration of BMC. These abnormalities were significantly reduced in CSD treated mice, indicating the therapeutic potentials of CSD as treatment targeting several aspects of heart failure.

## Materials and methods

### Animals

Wild-type C57BL/6J male mice (10–12 wk old) were purchased from Jackson Laboratory (Bar Harbor, Maine). All animal experiments were performed under a protocol approved by the Medical University of South Carolina (MUSC) Institutional Animal Care and Use Committee (IACUC).

### AngII infusion

Mini-osmotic pumps (ALZET 1002; DURECT Corporation, Cupertino, CA) were implanted during isofluorane anesthesia under the loose skin slightly posterior to the scapulae as described previously [46]. The pumps contained either 100 μl saline or AngII (2.1 mg/kg/day) and were designed to deliver their contents at 0.25 μl/h for 2 wk. CSD (amino acids 82–101 of caveolin-1, DGIWKASFTTFTVTKYWFYR-NH_2_) was purchased from Elim Biopharmaceuticals (Hayward, CA). CSD and vehicle treatments (100 μl of daily *i*.*p*. injections of CSD (50 μg/mouse) or vehicle (1% DMSO in water)) were initiated on the day of surgery. At the end of 2 wk, heart function was evaluated by echocardiography and the mice were sacrificed under deep anesthesia. Mice were systemically perfused via the left ventricle (LV) with PBS and heart and kidney samples were processed for Western blot, histochemical, and immunohistochemical studies.

### Echocardiography

Echocardiography (Echo) was performed at 2 wk after surgery using a Vevo2100 imaging system (VisualSonics, Toronto) as previously described [43, 44]. Heart rate was maintained at 400–500 bpm during isoflurane anesthesia. The 22–55 MHz linear transducer probe was used for two-dimensional B- and M-mode analyses. Offline analyses of M-mode images of the parasternal short-axis view at papillary level, was performed using 1.2.0 software to calculate ejection fraction (EF), LV mass (corrected), and posterior wall thickness in diastole (pWTh). B-mode images of the parasternal long axis were used to calculate cardiac output (CO) and stroke volume (SV), and end-diastolic volume (EDV) and end-systolic volume (ESV). Tissue Doppler imaging was used to measure velocity of posterior left ventricular (LV) wall motion at the papillary muscle level in short-axis view. 5 to 10 cycles were recorded to calculate both isovolumic relaxation time (IVRT) and isovolumic contraction time (IVCT). Blood pressure (BP) was measured on conscious mice using a computerized non-invasive CODA tail-cuff blood pressure system (Kent Scientific Corp., Torrington, CT) which automatically performs rapid, simultaneous measurements of both systolic and diastolic BP.

### Western blotting

Analyses were carried out as previously described [43, 44] with minor changes. Soluble and insoluble fractions of LV were prepared. Briefly, 50 mg LV or kidney tissue were homogenized using a T25 Ultra-Turrax homogenizer for 1 min on ice in 1 ml of radioimmunoprecipitation assay (RIPA) buffer (50 mM Tris-HCl (pH 7.4), 1% NP-40, 0.5% sodium deoxycholate, 150 mM NaCl, 0.1% sodium dodecyl sulfate and protease and phosphatase inhibitors). The homogenate was kept on ice for 15 min, then centrifuged at 10,000 g for 15 min. The soluble fraction was mixed with an equal volume of 2X Sample Buffer and boiled; the insoluble pellet fraction was suspended in 0.5 ml of 1X Sample Buffer and boiled and clarified. Samples were resolved by SDS-PAGE at 4°C (Invitrogen 4–12% Bis-Tris Gels, 1X MOPS buffer) and transferred to Invitrolon™ PVDF membranes (ThermoFisher Scientific (Invitrogen), Waltham, MA). Membranes were blocked with 5% milk protein, then incubated with primary antibodies overnight. After washing in Tris-buffered saline, the membranes were incubated with HRP-labeled secondary antibodies and target proteins detected by enhanced chemiluminescence.

#### Histochemistry and immunohistochemistry

Mouse heart and kidney tissue samples perfused as described above were fixed with 4% formaldehyde for 18 h, then dehydrated with ethanol and xylene washes for embedding in paraffin [43, 45]. To detect collagen deposition, tissue sections (7 μm thick) were stained with Picrosirius Red and viewed by polarized light microscopy. To detect HSP47, paraffin-embedded tissue sections were deparaffinized in an oven at 60° C for 1 h followed by rehydration in water. Antigen retrieval was performed using citrate buffer (10 mM citric acid, 0.05% Tween 20, pH 6.0) at 90°C-100°C in a water bath for 20 min. After cooling, slides were washed in water followed by PBS and then blocked with 10% Normal Donkey Serum in PBS for 1 h at room temperature in a humid chamber. Slides were incubated with HRP-labeled anti-HSP47 antibody (final concentration 2 μg/ml) in humid conditions at room temperature for 5 h. Slides were then washed with 1X PBS and stained with the nuclear stain DAPI at RT for 30 min. The slides were washed with PBS, mounted on coverslips and viewed using laser scanning confocal microscopy.

### Myocyte hypertrophy

In addition to measuring heart weight to body weight ratio, the extent of cardiac hypertrophy was analyzed by measuring myocyte cross-sectional area on hematoxylin-eosin stained sections. Hearts were fixed with 4% formaldehyde and embedded in paraffin. 7-μm thick LV sections were stained with hematoxylin-eosin and viewed using a 20X lens. The circumference of myocytes in five random fields was traced, providing a calculation of their cross-sectional area using SigmaScan Pro image analysis [47].

### BMC migration assay

To isolate bone marrow cells (BMC) for migration experiments, femurs and tibias are dissected, the end of the bones snipped off, and bone marrow cells flushed from the shafts with PBS pH 7.2/ 0.5% BSA/ 2 mM EDTA. The cells are then disaggregated by gentle pipetting, passed through a 40 μm cell strainer, washed by centrifugation in the same buffer, and counted. Flow cytometry showed that 65% of BMC were monocytes. Migration experiments are performed as described [48] using SDF-1 (100 ng/ml) as the chemoattractant. Cells that have migrated are counted in six high power fields per filter.

### Statistical analyses

Values are presented as mean ± SEM. Differences were analyzed between groups using one-way analysis of variance (ANOVA) followed by a *post hoc* Tukey’s multiple comparison to determine statistical significance.

## Results

### Suppression of cardiac hypertrophy by CSD in AngII-infused mice

**Fig 1:**
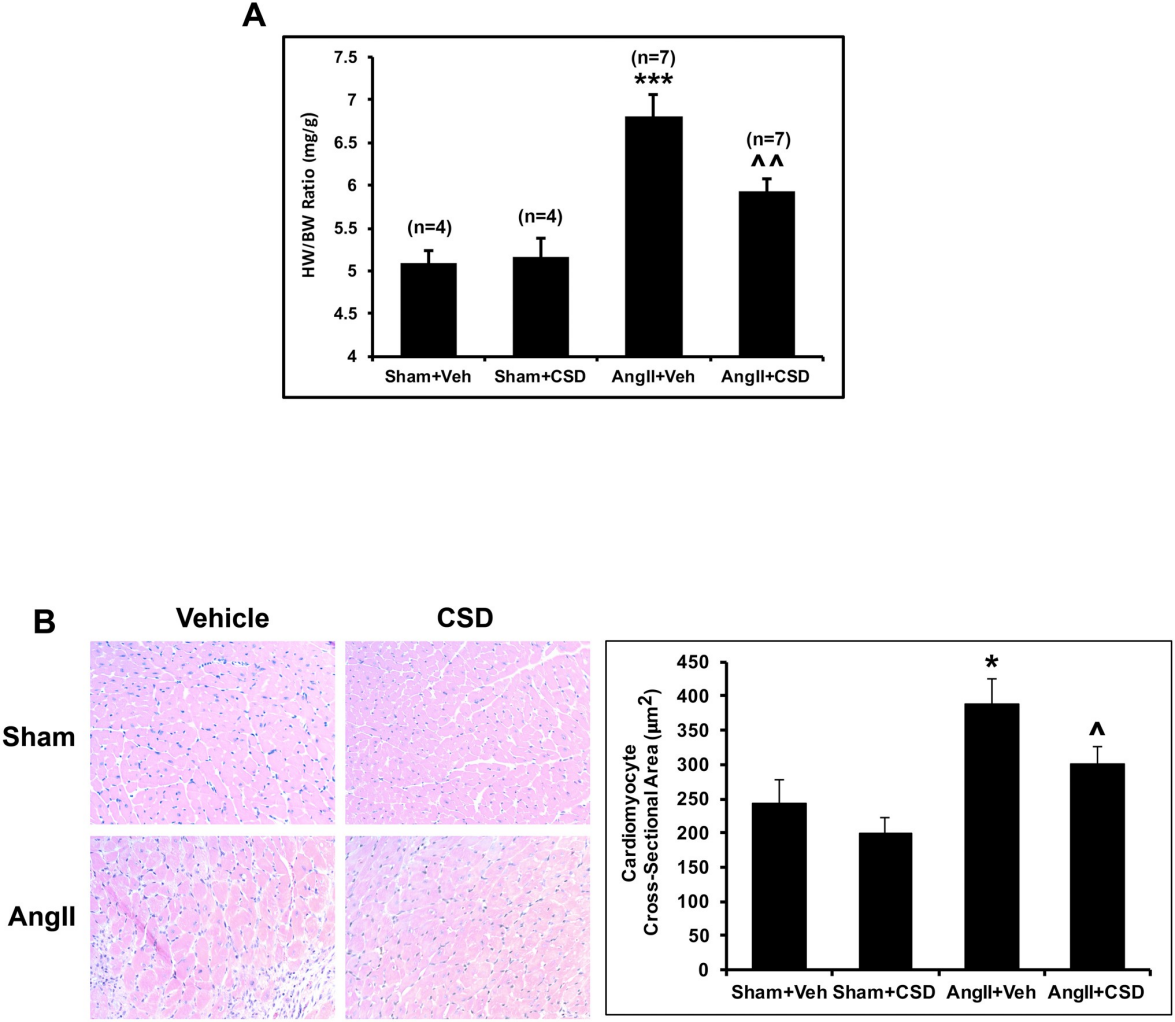
CSD reverses AngII-induced cardiac hypertrophy. Mice infused with AngII or saline for 2 wk received daily i.p. injections of CSD or vehicle. **(A)** Prior to sacrifice, body weight (BW) was measured. Heart tissue was weighed after sacrifice. HW to body weight ratio for each group of mice. **(B)** Cardiomyocyte cross-sectional area was calculated by staining LV tissue sections with hematoxylin-eosin. Quantitative analysis of cross-sectional area was determined by measuring at least 50 cardiomyocytes for each group (n = 3) using SigmaScan Pro image analysis. Statistical significance is shown as *p < 0.05 and ***p < 0.001 for Sham+Veh vs AngII+Veh and ^p < 0.05 and ^^p < 0.01 for AngII+Veh vs AngII+CSD.

Several studies show that continuous infusion of AngII *in vivo* increases cardiac mass and extracellular matrix (ECM) deposition. Therefore, we explored whether these changes induced by AngII infusion are suppressed in mice treated with CSD. We first determined heart weight to body weight ratio (HW/BW ratio) for: Sham mice treated with vehicle (Sham+Veh), Sham mice treated with CSD (Sham+CSD), AngII mice treated with vehicle (AngII+Veh), and AngII mice treated with CSD (AngII+CSD). Compared to Sham+Veh mice, AngII+Veh mice showed a highly significant increase in HW/BW ratio (Fig 1A). CSD treatment suppressed this effect. As expected, CSD did not affect HW/BW ratio in sham mice. The change in HW/BW ratio was entirely due to changes in heart weight as significant changes did not occur in body weight among the four groups.

To further confirm that CSD suppresses AngII-induced myocyte hypertrophy, we measured cardiomyocyte cress-sectional area. Compared to Sham mice, AngII infusion caused a significant increase in myocyte cross-sectional area (Fig 1B). While CSD treatment in Sham mice show no appreciable change, it significantly suppressed cardiomyocyte cross-sectional area in AngII infused mice.

### Suppression of cardiac and renal fibrosis by CSD in AngII-infused mice

**Fig 2:**
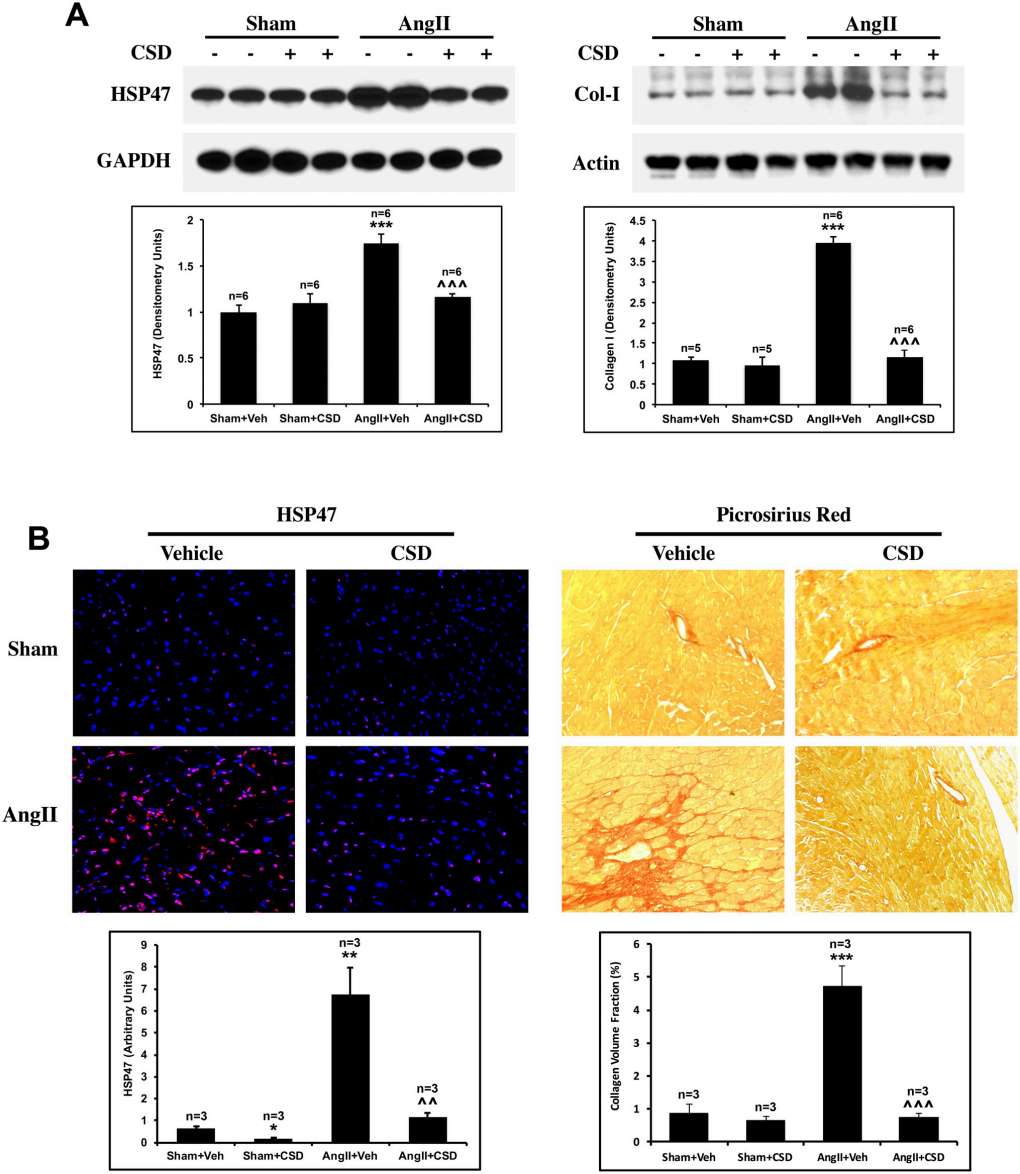
CSD reverses AngII-induced cardiac fibrosis. Mice infused with AngII or saline for 2 wk received daily i.p. injections of CSD or vehicle. **(A)** RIPA buffer-solubilized (left panel) and sample buffer-solubilized (right panel) protein samples from LV tissue were prepared as described in the Methods and used in Western blotting experiments for HSP47, Col I, and the indicted loading controls. Graphs show quantitation of Western blots from 4 independent mice for each group. Statistically significance is shown as ***p < 0.001 for Sham+Veh vs AngII+Veh and ^^^p < 0.001 for AngII+Veh vs AngII+CSD. (**B**) Histochemical analyses: Left: LV tissue sections from the indicated mice were stained with anti-HSP47 (red), and with DAPI (blue) to detect nuclei. The graph quantifies total HSP47 staining intensity per field in arbitrary units. Three or four mice per category were used and at least four randomly selected fields for each mouse were used for quantitation. Right: LV tissue sections were stained with Picrosirius Red to detect collagen. The graph quantifies collagen volume fraction, calculated from photomicrographs using SigmaScan Pro-5. Three or four mice per category were used and at least four randomly selected fields for each mouse were used for quantitation. **p<0.01, ***p < 0.001 for AngII+Veh vs Sham+Veh; ^^p<0.01, ^^^p < 0.001 for AngII+CSD vs AngII+Veh.

We evaluated cardiac fibrosis in terms of the levels of collagen I and the collagen chaperone HSP47 in LV samples. (Fig 2). We previously showed that in the TAC model, HSP47 and collagen I were increased and that these changes were suppressed in CSD treated mice [43]. Similar findings were observed in AngII-treated mice in the present study. Western blot analyses (Fig 2A) clearly showed significant increases in HSP47 and collagen levels in AngII-treated mice that were effectively blocked by CSD. IHC also showed a robust increase in the number of HSP47-positive cells in AngII-infused mice treated with vehicle (Fig 2B, left panel) that was reduced to the Sham baseline level when mice were treated with CSD. Similarly, Picrosirius Red staining showed a robust increase in collagen deposition both in the perivascular and interstitial areas in AngII treated mice that was decreased to baseline with CSD treatment (Fig 2B, right panel).

**Fig 3:**
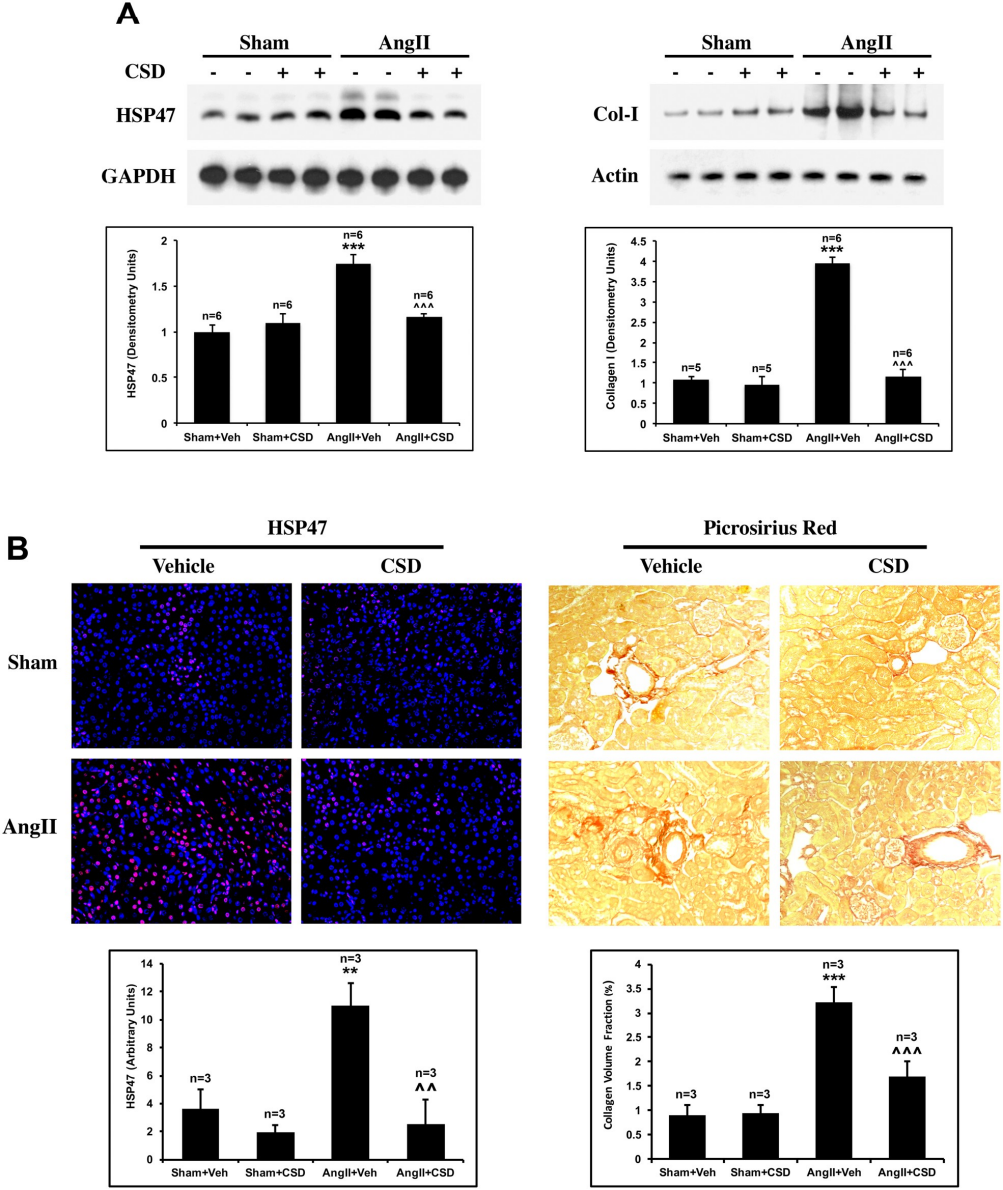
CSD reverses AngII-induced kidney fibrosis. Mice infused with AngII or saline for 2 wk received daily i.p. injections of CSD or vehicle. **(A)** RIPA buffer-solubilized (left panel) and sample buffer-solubilized (right panel) protein samples from kidney tissue were prepared as described in the Methods and used in Western blotting experiments for HSP47, Col I, and the indicted loading controls. Graphs show quantitation of Western blots from 4 independent mice for each group. Statistically significance is shown as ***p < 0.001 for Sham+Veh vs AngII+Veh and ^^^p < 0.001 for AngII+Veh vs AngII+CSD. (**B**) Histochemical analyses: Left: Kidney tissue sections from the indicated mice were stained with anti-HSP47 (red), and with DAPI (blue) to detect nuclei. The graph quantifies total HSP47 staining intensity per field in arbitrary units. Three or four mice per category were used and at least four randomly selected fields for each mouse were used for quantitation. Right: Kidney tissue sections were stained with Picrosirius Red to detect collagen. The graph quantifies collagen volume fraction, calculated from photomicrographs using SigmaScan Pro-5. Three or four mice per category were used and at least four randomly selected fields for each mouse were used for quantitation. **p<0.01, ***p < 0.001 for AngII+Veh vs Sham+Veh; ^^p<0.01, ^^^p < 0.001 for AngII+CSD vs AngII+Veh.

AngII infusion and the associated development of systemic hypertension affect multiple organs, in particular heart and kidney [2–4, 28, 49, 50]. Therefore, we explored whether fibrosis could be also observed in the kidney following the 2-wk AngII infusion period, and whether CSD treatment suppresses the development of kidney fibrosis. Similar to our findings with the heart samples, Western blot analyses kidney samples showed a substantial increase in HSP47 and collagen I levels in AngII+Veh mice that was significantly reduced in AngII+CSD mice (Fig 3A). Sham+Veh and Sham+CSD mice showed similar levels of collagen I and HSP47. These results were validated for HSP47 by IHC and for collagen I by Picrosirius Red staining (Fig 3B). IHC studies for HSP47 showed low levels of HSP47 in Sham+Veh mice (Fig 3B, left panel). AngII infusion resulted in a substantial increase in HSP47 levels that was spread evenly in the tissue. CSD treatment blocked significantly the HSP47 increase in AngII infused mice. Similarly, Picrosirius Red staining (Fig 3B, right panel) showed a substantial increase in ECM deposition that was present mostly in the perivascular area. This AngII-induced ECM deposition was significantly suppressed in CSD treated mice.

### Suppression of profibrotic signaling by CSD

**Fig 4:**
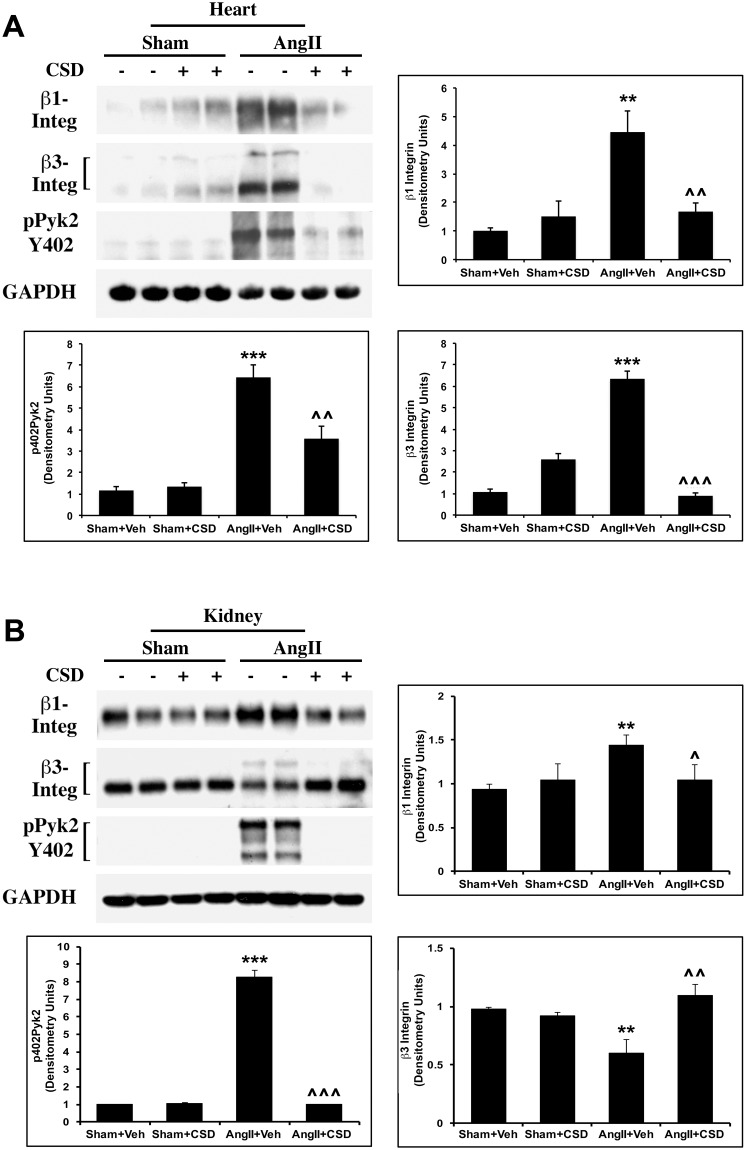
CSD inhibits AngII-induced increases in integrin levels and NTK activation in the heart and kidney. Mice were infused with AngII or saline for 2 wk and received daily i.p. injections of CSD or vehicle. (**A**) RIPA extracts of LV tissue were used in Western blotting experiments with antibodies against β1 and β3 integrins, phospho-Pyk2-Y402, and GAPDH (loading control). Graphs show quantitation of Western blots using 4 independent mice for each group. Statistically significant changes are shown as **p < 0.01, ***p < 0.001 for Sham+Veh vs AngII+Veh and ^^p < 0.01, ^^^p < 0.001 for AngII+Veh vs AngII+CSD. (**B**) RIPA extracts of kidney tissue were used in Western blotting experiments with antibodies against β1 and β3 integrins, phospho-Pyk2-Y402, and GAPDH (loading control). Graphs show quantitation of Western blots using 4 independent mice for each group. Statistically significant changes are shown as **p < 0.01, ***p < 0.001 for Sham+Veh vs AngII+Veh and ^p < 0.05, ^^p < 0.01, ^^^p < 0.001 for AngII+Veh vs AngII+CSD.

We have previously shown that β1 and β3 integrin and the tyrosine kinase Pyk2 regulate collagen expression in PO mouse myocardium [44, 45]. The enhanced expression of these integrins and the activation of Pyk2 in the TAC model are suppressed by CSD [43]. Similarly, AngII caused a significant increase in β1 and β3 integrin levels (both the 90 kD and 130 kD isoforms) and Pyk2 activation (Tyr-402 phosphorylated Pyk2) in the heart that was blocked in CSD treated mice (Fig 4A). As fibrosis was observed in both heart and kidney of AngII-infused mice, we also analyzed whether there are changes in the levels of these signaling proteins in the kidney (Fig 4B). Indeed, AngII infusion increased β1 integrin level and activated Pyk2 in the kidney and these effects were suppressed by CSD. The results obtained with β3 integrin were more complex. While AngII decreased the levels of the major 90 kD isoform, it increased the levels of 130 kD high molecular weight β3 integrin (Fig 4B) which we previously showed to be phosphorylated. Both the decrease in the major isoform and the increase in the high molecular weight isoform were suppressed by CSD.

**Fig 5:**
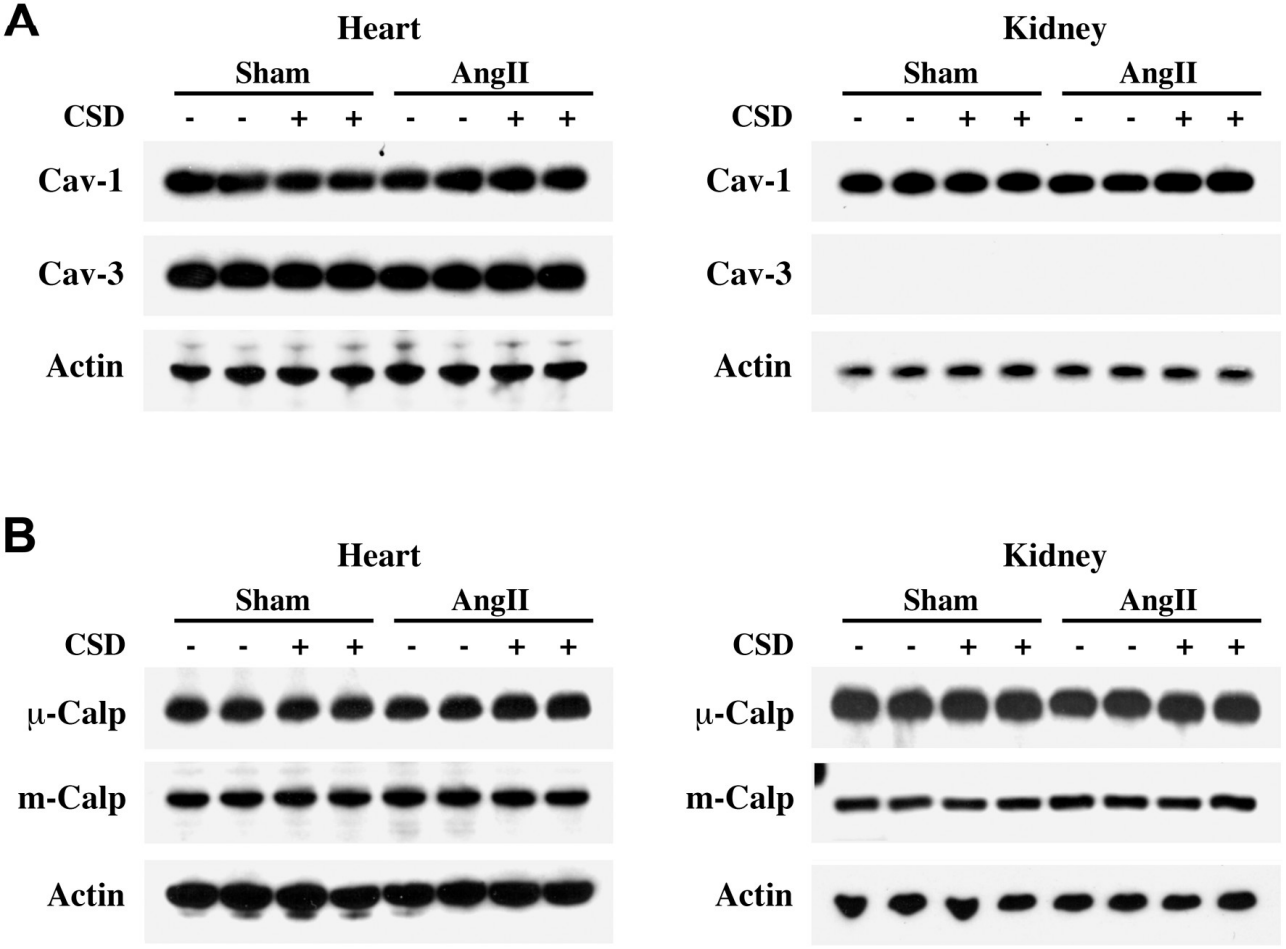
Effect of AngII-infusion and CSD treatment on caveolin and calpain isoforms in the heart and kidney. Mice were infused with AngII or saline for 2 wk and received daily i.p. injections of CSD or vehicle. Extracts were prepared as described in the previous figure legends. (**A**) RIPA extracts of LV and kidney tissues were used in Western blotting experiments with antibodies against caveolin-1 (cav-1), caveolin-3 (cav-3) and β-actin (loading control). (**B**) RIPA extracts of LV tissue and kidney tissues were used in Western blotting experiments with antibodies against μ-calpain (μ-Calp), m-calpain (m-Calp), and β-actin (loading control). No statistically significant effects of AngII or CSD were observed.

We also analyzed caveolin and calpain levels in the heart and kidney. Both caveolin-1 and the muscle specific isoform caveolin-3 were expressed in the heart whereas only caveolin-1 was detected in the kidney (Fig 5A). Because calpains have been shown to be downstream mediators of cardiovascular remodeling in a 4-wk AngII infusion study [51], we quantified the levels of the μ- and m-calpain in the heart and kidney (Fig 5B). Neither AngII nor CSD, either alone or together, showed a significant effect on the levels of any caveolin or calpain isoform in the heart or kidney.

### Improvement of cardiac function by CSD in AngII treated mice

Chronic AngII infusion causes multiple changes including ventricular wall thickening, fibrosis, and compromised ventricular function [3, 4, 28, 49, 50]. When we compared echocardiographic measurements for the four groups of mice (Table 1), AngII+Veh mice showed several pathological effects compared to Sham+Veh mice including a significant loss in LV function with reduced levels of EF, FS, CO, and SV and increased IVRT, but not IVCT.

**Table 1:** CSD reverses AngII-induced hypertrophy and deficits in ventricular function.

	Sham (n = 4)	Sham+CSD (n = 4)	AngII+Veh (n = 7)	AngII+CSD (n = 6)
**SAX M mode**				
EF (%)	56.12 ± 2.0	56.67 ± 1.5	44.23 ± 3.3*	58.86 ± 1.68^^
FS (%)	29.03 ± 1.3	29.05 ± 1.1	21.45 ± 1.8**	30.26 ± 1.21^^
LV Mass (mg)	124.1 ± 7.6	104.2 ± 11	143.9 ± 6.2*	110.4 ± 8.4^^
pWTh (mm)	0.92 ± 0.05	1.00 ± 0.04	1.27 ± 0.06**	1.13 ± 0.05^
**PSLAX B Mode**				
CO (ml/min)	16.29 ± 1.40	13.59 ± 0.44	12.03 ± 0.80**	14.61 ± 1.11^
EF (%)	57.83 ± 1.48	57.2 ± 1.46	46.36 ± 2.65**	53.6 ± 2.28^
SV (μl)	36.33 ± 1.63	32.8 ± 1.81	24.98 ± 1.67***	32.52 ± 2.09^^
EDV (μl)	62.74 ± 1.64	57.6 ± 4.31	54.91 ± 4.30	60.39 ± 1.72
ESV (μl)	26.41 ± 0.81	24.8 ± 2.59	29.92 ± 3.47	27.87 ± 0.92
**Tissue Doppler**				
IVCT (ms)	13.73 ± 0.60	13.14 ± 0.80	13.07 ± 0.88	12.26 ± 0.46
IVRT (ms)	20.27 ± 0.59	17.76 ± 1.47	27.19 ± 2.32*	21.85 ± 1.33^

M-mode echocardiographic measurements in parasternal short-axis (PSAX) view were used to quantify changes in LV mass, pWTh (in diastole), EF, and FS. B-mode measurements in parasternal long-axis (PSLAX) view were used to quantify changes in CO, EF, SV, EDV and ESV. Tissue Doppler measurements in PSAX view were used to measure IVRT and IVCT. Values are shown as Mean ± SEM. Statistically significant changes are shown as *p < 0.05, **p < 0.01 and ***p < 0.001 for Sham+Veh vs AngII+Veh and ^p < 0.05 and ^^p < 0.01 for AngII+Veh vs AngII+CSD.

Further, pWTh at diastole was significantly increased. These pathological effects were all inhibited in AngII+CSD mice. In addition, AngII decreased EDV and this effect was also suppressed by CSD, but these changes did not achieve statistical significance. Compared to the Sham+Veh group, Sham+CSD mice showed no significant changes in any parameter examined.

Measurements were performed to determine whether the beneficial effects of CSD might be downstream from effects on BP. In fact, as well known, AngII significantly increased BP. However, this effect was not at all suppressed by CSD (Table 2), indicating that the various beneficial effects by CSD are not related to effects on BP.

**Table 2:** CSD does not affect AngII-induced blood pressure (BP) increase.

	Sham+Veh	AngII+Veh	AngII+CSD
BP	(n = 5)	(n = 9)	(n = 6)
DBP mmHg	117 ± 4.4	150 ± 10*	147 ± 9.8
SBP mmHg	149 ± 5.21	183 ± 10*	182 ± 10.2

BP was measured on conscious mice using a computerized non-invasive CODA tail-cuff blood pressure system (Kent Scientific Corp., Torrington, CT) which automatically performs both systolic and diastolic BP. Values are shown as Mean ± SEM. Statistically significant changes are shown as *p < 0.05 for Sham+Veh vs AngII+Veh.

### CSD suppresses AngII-induced hypermigration of bone marrow cells (BMC) and hyper-permeability of heart and kidney vasculature

**Fig 6:**
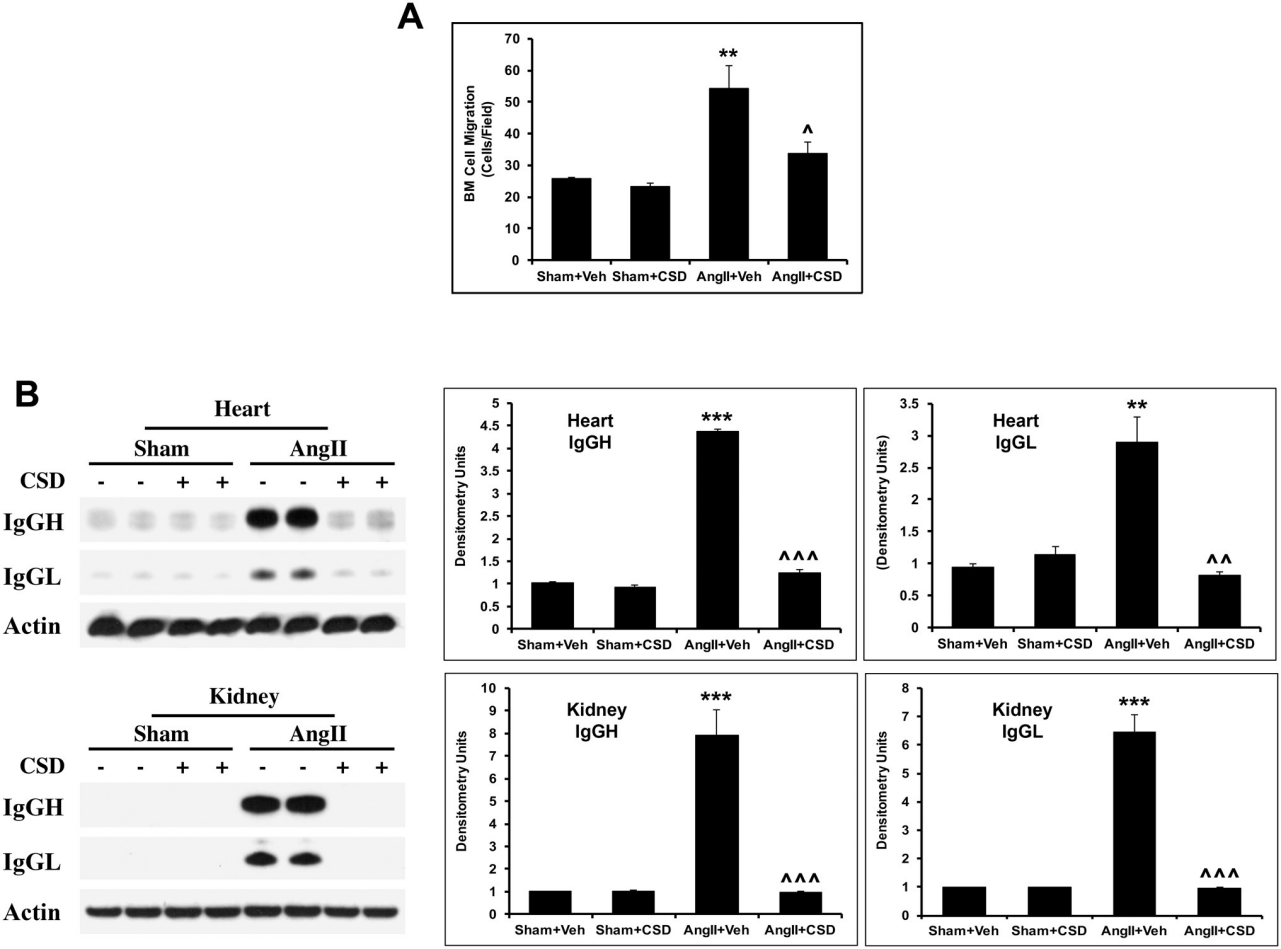
CSD suppresses both AngII-induced hypermigration of bone marrow cells (BMC) and AngII-induced vascular leakage of IgG into heart and kidney tissues. Mice were infused with AngII or saline for 2 wk and received daily i.p. injections of CSD or vehicle. (**A**) Migration experiments were performed as described in the Methods using isolated BMC. Cells that migrated towards SDF-1 were counted in six high power fields per filter. Graphs show quantitation of migrated cells using three independent mice for each group. Statistically significant changes are shown as **p < 0.01 for Sham+Veh vs AngII+Veh and ^p < 0.05 for AngII+Veh vs AngII+CSD. (**B**) RIPA extracts of LV and kidney tissue were used in Western blotting experiments. HRP-conjugated anti-mouse secondary antibody with no primary was used to detect mouse IgG. (Left) Typical Western blot results from LV and kidney tissue showing IgGH (IgG heavy chain) and IgGL (IgG light chain). (Right) Quantification of IgGH and IgGL (n = 4). Statistically significant changes are shown as **p < 0.01, ***p < 0.001 for Sham+Veh vs AngII+Veh and ^^p < 0.01, ^^^p < 0.001 for AngII+Veh vs AngII+CSD.

Earlier studies have shown that CSD suppresses the hypermigration of BMC in a mouse model of lung fibrosis [35, 36, 39, 52]. To determine whether BMC from AngII-infused mice also have a hypermigratory phenotype and whether this phenotype is suppressed by CSD treatment in vivo, we isolated BMC from mouse femurs, and used them in migration experiments with SDF-1 as the chemoattractant. AngII infusion caused a significant increase in BMC migration while CSD treatment almost completely suppressed the enhanced migration (Fig 6A). CSD did not affect the basal migration of BMC in mice treated with saline vehicle.

Vascular hyperpermeability (leakage) occurs due to AngII treatment [53, 54]. We evaluated whether this effect was suppressed when mice were treated with CSD by performing Western blot analysis of IgG levels in heart and kidney tissue. Our studies show a robust release of IgG both into the heart and kidney of AngII treated mice (Fig 6B) that was almost completely suppressed by CSD treatment in vivo.

## Discussion

We recently used a murine TAC model to induce LV hypertrophy and fibrosis by PO and showed that CSD treatment can block myocardial fibrosis and improve ventricular function [43]. In addition, the beneficial effects of CSD were found to be accompanied by reduced expression of collagen I, HSP47 (a collagen chaperone protein), and integrins (β1 and β3), and by the reduced activation of NTKs (Pyk2 and c-Src). To test the antifibrotic effect of CSD in an independent model, here we have used AngII infusion, a common model for inducing heart and renal disease [5, 19, 29, 55–57]. These studies, besides providing a second model for heart failure and a model for kidney disease, have expanded our knowledge of the cell types involved in the beneficial effects of CSD on heart failure.

In the present study, we show several beneficial effects of CSD in AngII-infused mice: (i) CSD suppressed AngII-induced fibrosis (evaluated in terms of Col I and HSP47 levels) in the heart and kidney, (ii) CSD suppressed AngII-induced morphological changes in the heart (evaluated in terms of pWTh, cardiac mass, and myocyte hypertrophy), (iii) CSD suppressed the usual AngII-induced pathological changes in ventricular function (EF, FS, SV, CO, IVRT) as determined by echocardiography, (iv) CSD suppressed the AngII-induced hypermigratory behavior of BMC towards SDF-1, and (v) CSD suppressed AngII-induced vascular leakage of IgG into the heart and kidney.

Multiple cell types appear to contribute to the beneficial effects of CSD (Table 3). Fibroblasts and monocytes from patients and mice with fibrotic disease have been found to be deficient in caveolin-1 [35, 36, 48]. The deficiency in fibroblasts results in their overexpression of Col I and results in organ fibrosis [58, 59]. In addition, the development of lung, skin, and cardiac fibrosis occurs in caveolin-1 null mice due to effects involving fibroblasts and M2 macrophages [33, 34, 37, 38]. The deficiency in monocytes leads to their enhanced expression of chemokine receptors resulting in hypermigration toward the cognate ligands for these receptors in an in vitro assay [48]. In vivo, this enhanced migration is observed as the enhanced recruitment of monocytes into stressed organs. These effects of caveolin-1 deficiency in fibroblasts and monocytes are suppressed by CSD (Figs 2, 3 and 6A and Table 3), strongly suggesting that in these cells CSD is acting as a caveolin-1 surrogate.

**Table 3:** Cell type specific mode of action of CSD.

Cell Types	Caveolin levels		CSD Action
	Baseline	Fibrosis	
Fibroblasts	Moderate Cav-1	Decrease	Surrogate
Monocytes	Low Cav-1	Decrease	Surrogate
Endothelial cells	High Cav-1	No Change	Competitor
Cardiomyocytes	High Cav-3	No Change	Competitor

Recruited monocytes can contribute to fibrosis by differentiating into macrophages that secrete factors that promote resident fibroblasts to become myofibroblasts [2, 6, 13, 14]. Some of these secretory factors include vasoactive agents (angiotensin, endothelin), growth factors (PDGF, TGF-β, FGF, etc.), hormones (aldosterone, corticosterone), and cytokines (interleukins). These agents in turn promote fibroblast proliferation and ECM secretion via a mechanism involving integrins [49, 56, 60]. Recruited monocytes may also serve as direct myofibroblast precursors. Monocytes differentiate into CD45+/ Col I+ fibroblast-like cells (often referred to as fibrocytes) that express the myofibroblast marker ASMA and contribute to fibrosis [5, 16–26]. The differentiation of monocytes into myofibroblastic cells is enhanced when their caveolin-1 levels are depressed, but is suppressed by CSD, again indicating that CSD is acting as a caveolin-1 surrogate [34–36, 39, 52, 61].

AngII infusion increases ROS levels by activing NADPH oxidase [62], resulting in endothelial damage and leakage of plasma proteins into tissues [53, 54]. Our present data clearly show leakage of IgG into both the heart and kidney, suggesting that other serum proteins are also released into the tissues including growth factors and cytokines that will alter cell behavior. Importantly, CSD treatment of AngII-infused mice completely blocked IgG leakage, consistent with the idea that reversal of vascular leakage by CSD is one major cause of its beneficial effects.

This beneficial effect of CSD in endothelial cells appears to involve CSD functioning as a competitor of caveolin-1 rather than as a surrogate (Table 3). Caveolin-1 is expressed at high levels in vascular endothelial cells. Caveolin-1 deletion prevents AngII-induced vascular abnormalities [63]. We find that CSD prevents AngII-induced vascular leakage (Fig 6B). For caveolin-1 KO animals and wild-type animals treated with CSD to show a similar phenotype, it must be that CSD is acting as a competitor of caveolin-1 in certain cell types, rather than as a surrogate. This is not a novel concept in that similar observations have already been reported on the function of caveolin-1 in endothelial cells [64–66]. Indeed, in one of these studies, CSD was shown to inhibit VEGF-induced vascular leakage [66].

**Fig 7:**
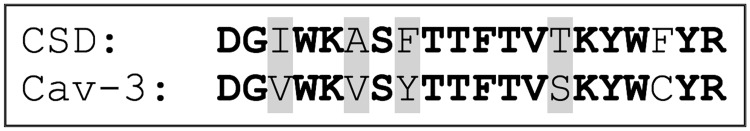
Comparison of CSD sequences. Homologous amino acids in the CSD region of caveolin-1 and caveolin-3 are shown in bold letters; amino acids with similar properties are highlighted in grey.

In AngII-infused mice, the increased cardiac mass is primarily due to myocyte hypertrophy rather than fibrosis. Our findings show that CSD suppresses the AngII-induced increase in heart weight, ventricular wall thickness, and cardiomyocyte cross-sectional area (Fig 1 and Table 1). Interestingly, although caveolin-1 is expressed in multiple cell types both in the heart and kidney, the muscle specific isoform caveolin-3 is expressed only in cardiomyocytes in the heart and is not expressed in the kidney (Fig 5A). Caveolin-3 contains a sequence highly homologous to CSD (Fig 7). Therefore, the decrease in the myocyte cross-sectional area that we observed when AngII mice were treated with CSD (Fig 1) may result from the effect of CSD on caveolin-3 signaling [67].

At the molecular level, CSD has been shown to affect several signaling mechanisms, including G-protein and MEK/ERK signaling [52, 68, 69]. Our earlier studies show the importance of integrins, in particular β3 integrin, and the subsequent activation of NTKs for the development of cardiac fibrosis in PO myocardium [44, 45, 47]. Furthermore, we showed that CSD could block these changes in integrin expression and NTK activation [43]. Other laboratories have shown the importance of β3 integrin and NTK activation for profibrogenic signaling [49, 56, 60, 70]. In the present study, we show that AngII infusion, similar to TAC, caused an enhanced expression of β1 and β3 integrins and Pyk2 activation in the heart and kidney that were blunted by CSD treatment (Fig 4). These data strongly suggest that CSD has a direct effect on profibrotic signaling in fibrotic tissue.

Our studies have also addressed whether AngII and CSD affect the levels of caveolin itself and of calpains which have been shown to be regulated in the kidney during 4 wk of AngII treatment [51]. Our studies on caveolin levels show that caveolin-1 and caveolin-3 are expressed in the heart whereas only caveolin-1 is expressed in the kidney. However, their levels were not found to be altered by AngII and, as expected, they were also not affected by CSD. We observed no significant changes in calpain levels (neither m-calpain nor μ-calpain) in the heart or kidney due to a 2 wk infusion of AngII or to CSD (Fig 5).

Our echocardiographic measurements using AngII model confirm our previous results in the TAC model [43]. The effects we observed on heart function and morphology due to AngII infusion are consistent with the literature [71–73], although some laboratories showed only significant changes in cardiac mass and fibrosis, but not in cardiac function (EF, SV and CO) [5, 74]. These differences could be due to variations in AngII doses [75]. Finally, the increased IVRT that we observe in AngII-infused mice suggests compromised diastolic relaxation due to ventricular stiffness caused by fibrosis and myocyte hypertrophy. Therefore, it follows that the suppression of fibrosis and myocyte hypertrophy by CSD would reverse the increase in IVRT caused by AngII.

We measured BP to explore whether CSD affects the well-known AngII-induced increase in systolic and diastolic BP. Our studies show that, as expected, AngII infusion caused BP to increase. However, this increase was not affected by CSD Table 2. Consistent with our observations, caveolin-1 deletion or CSD treatment does not appear to exhibit major effects on BP: (i) Caveolin-1 silencing in mice does not affect the basal or the AngII-induced increase in BP [63], although low level changes are reported in another study [76]. (ii) In the long-term, AngII inhibits eNOS [77] thereby impairing vasorelaxation and causing increased BP. CSD treatment does not reverse the inhibitory effect of AngII on eNOS. Rather, CSD suppresses eNOS activation [66, 78]. We also showed previously that CSD suppresses TAC-induced eNOS activation [43]. In summary, these studies support the concepts that CSD does not suppress the AngII-mediated increase in BP and that therefore the beneficial effects of CSD in AngII-infused mice must be independent of changes in BP.

In conclusion, our studies clearly show that CSD suppresses fibrosis and vascular leakage in the heart and kidney following AngII infusion in mice. Furthermore, the AngII-induced increase in cardiac hypertrophy and compromised ventricular function are reduced substantially when mice are treated with CSD during AngII infusion. These data strongly indicate the therapeutic potentials of CSD for patients with hypertensive heart and kidney diseases.

## Abbreviations

ACE: angiotensin-converting enzyme
AngII: angiotensin II
AT1: angiotensin receptor 1
BP: blood pressure
CHF: congestive heart failure
CO: cardiac output
CSD: caveolin-1 scaffolding domain
ECM: extracellular matrix
EF: ejection fraction
HSP47: heat shock protein-47
HW/BW: heart weight to body weight ratio
IHC: immunohistochemistry
IVRT: isovolumic relaxation time
LV: left ventricle
LVH: LV hypertrophy
Nox: NADPH oxidase
NTKs: nonreceptor tyrosine kinases
PO: pressure overload
pWTh: posterior wall thickness in diastole
ROS: Reactive oxygen species
SDF1: stromal-derived factor-1
SV: stroke volume
TAC: transverse aortic constriction
